# Complementary and alternative medicine utilisation in NHS and private clinic settings: a United Kingdom survey of 400 infertility patients

**DOI:** 10.1186/1743-1050-2-5

**Published:** 2005-04-04

**Authors:** Catherine Coulson, Julian Jenkins

**Affiliations:** 1 Centre for Reproductive Medicine, Department of Obstetrics and Gynaecology, University of Bristol, St Michael's Hospital, Bristol BS2 8EG, UK

## Abstract

Some evidence suggests that complementary and alternative medicine (CAM) has found increased utilisation among patients seeking infertility treatment, although there is little information available to quantify this phenomenon. This is important information as there is marketing for CAM directed to this group and professionals need to be aware of the treatments their patients are receiving. Patients attending for infertility diagnosis and treatment often ask the physician about CAM; this paper seeks to compare the prevalence of CAM use among infertility patients in National Health Service (NHS) and private clinics. This paper provides results of a survey of couples (*n *= 400) divided equally between NHS and private settings. Our data suggest a high use of CAM particularly among female private patients, although patients appear sceptical of the efficacy of such treatment which is consistent with the literature.

## Introduction

Infertility patients are a vulnerable group that often seek a non-medical solution for their failure to conceive. The Cochrane Collaboration's definition of Complementary and Alternative Medicine (CAM) is "a broad domain of healing resources that encompasses all health systems, modalities, and practices and their accompanying theories and beliefs other than those intrinsic to the politically dominant health systems of a particular society or culture in a given historical period". This survey sought to compare use of CAM by infertile couples in NHS and private settings.

## Methods

The University of Bristol operates clinics in two settings: one is an NHS reproductive medicine clinic based at St. Michael's Hospital which treats ~500 new couples each year. Patients are seen for routine investigation and treatment of infertility, but no assisted conception is provided at this facility. The other clinic is a non-NHS office at the Centre for Reproductive Medicine, where patients are seen for private investigation and treatment of their infertility including in vitro fertilisation and donor insemination. This private service provides between 500–600 cycles of assisted conception and ~220 cycles of donor insemination each year. Between February and April 2001, all patients (men and women) attending a routine visit at either center were invited to complete an anonymous questionnaire regarding the patient's use of CAM (see additional file 1). The invitation was offered until 200 patients in each location had completed the survey. Although couples often attend together, men and women were approached separately. Each study participant completed only one questionnaire. Fishers exact test was used for comparison of proportions between groups.

## Results

The survey was simple to complete and the receptionist asked the patients for their replies at the end of the appointment, hence response rates in both services were high: 181 respondents from 200 NHS patients (124 women and 57 men) and 157 respondents of 200 private patients (120 women and 37 men), or 83% overall.

While these clinics only treat couples, for many of the routine visits it is the female partner who comes unaccompanied for procedures such as ultrasound scanning, post coital testing and donor insemination, thus explaining the higher frequency of women responders. Mean age of respondents was 36 and 34 for men and women in the private setting, respectively, and 39.8 and 35.5 in the NHS setting. Overall, in the private sector 13% of men and 40% of women had used CAM for their infertility compared with 12% of men and 23% of women in the NHS clinic (see table). It was also noted that overall 19% of patients had used CAM for other health problems in the past

Of patients who had used CAM, 10% thought it had been helpful for infertility, 13% felt it had helped them psychologically and that they had done everything possible, and 22% felt it had helped them to relax. In the space offered for additional comments (free text), several respondents indicated that they would like advice from their doctor specifically about CAM and infertility.

## Discussion and Conclusion

Infertility patients in our survey accessed CAM for their infertility more frequently than the overall use of CAM in the general population estimated by telephone and postal surveys. In 1999, a BBC telephone poll of 1204 randomly selected British adults were asked about the use of CAM in the preceding year [1]. Although the poll did not ask whether CAM had been accessed via a practitioner or by over the counter sales, it did reveal that 20% responders had accessed CAM over the preceding year. The most commonly used CAM was herbalism (34%), followed by aromatherapy (21%), homeopathy (17%), acupuncture (14%), reflexology (6%) and massage (6%). A postal survey of 5,010 randomly selected adults (53% response rate) showed that 13.6% had visited a CAM therapist in the preceding 12 months and 28.3% had bought an over the counter remedy and / or seen a CAM therapist [2]. As the use of CAM in the past by the infertile patients in our survey was consistent with the above reports, it seems reasonable to suggest that infertile patients in general make a greater use of CAM for their infertility than the general population.

We observed the highest use of CAM among women from the private clinic (40%). This may reflect a greater ability of these couples to afford the cost of CAM, even though they have the additional cost of their fertility treatment. There are various forms of CAM that may offer different things to different people. Although the CAM therapies used by the infertility patients are presented in table 1, there are many others including: acupressure, chiropractor, naturopathy, cranial osteopathy, osteopathy, Alexander technique, environmental medicine, kinesiology, Reiki, anthroposophic medicine, aromatherapy, autogenic training, visualisation, shiatsu, ayurveda massage, therapeutic touch, mediation and yoga.

**Table 1:** Use of complementary medicine for infertility as measured by questionnaire among NHS and private clinic patients (*n *= 400).

	NHS Male *n *= 57	NHS female *n *= 124	Private male *n *= 37	Private female *n *= 120
nutritional advice	2 (0.3)	9 (7)	2 (5)	21 (18)
Reflexology	0	11 (9)	1 (3)	19 (16)
Acupuncture	1 (0.2)	10 (8)	1 (3)	15 (13)
traditional Chinese medicine	0 (0.3)	5 (4)	1 (3)	6 (5)
herbalism	2 (0.3)	6 (5)	0	6 (5)
hypnosis	0	1 (1)	0	5 (4)
spiritual healing	2 (0.3)	5 (4)	0	2 (2)

CAM (all types)	7 (12)	29 (23)	5 (13)^a^	48 (40)^b^

Note: data presented as patient number (%)

^a^CAM use by females at private clinic vs. males at private clinic, *p *< 0.01 by Fishers exact test.

^b^CAM use by females at private clinic vs. females at NHS, *p *< 0.01 by Fishers exact test

A search of the CISCOM Complementary Medicine database (see ) revealed 105 reporting on different types of CAM for infertility and miscarriage. However, the studies were generally of a poor quality with no study providing prospective randomised controlled evidence of clinical efficacy for any form of CAM to improve the prognosis for infertile couples. Given this paucity of data, there is great need for properly conducted and appropriately controlled research in this area. Objective evaluation of the effectiveness of CAM in infertility would help to answer the questions that prompted this survey, *i.e*., is CAM effective, and how many people are using it? It is important that physicians be familiar with and consider CAM; patients should also understand the importance of evaluating efficacy of any intervention, including CAM.

It is interesting to speculate why infertility patients would access CAM despite the lack of evidence of efficacy. Indeed, the patients in our study population registered scepticism regarding CAM themselves. Apart from pregnancy, there are other valuable aspects to any infertility treatment. Quality of life measures and sense of well-being are valid, especially in a disability which may become chronic, such as infertility. This condition has a deeply distressing impact on how a woman or man feels about her/himself at the level of core identity. Patients often describe an encounter with a CAM provider in terms of a someone who was "really interested", a person "who listened really carefully to what I was saying" and "who seemed to understand how I feel". From this, it may be offered that traditional doctors would benefit by refining listening and counselling skills – an integral part of training, especially in general practice and now recognised as a core skill in RCOG and other specialist training. These skills can be enhanced by Balint groups and seminars offered by the Institute of Psychosexual Medicine, as well as formal training in psychotherapy.

While counselling can address some of these issues, doctors should strive to listen more carefully to patients so the consultation experience is satisfying and possibly, in itself, therapeutic. The effect of laying on of hands should not be underestimated; as physicians, we have the privilege of examining the bodies as well as the minds of our patients.

Although it is important to ensure the treatment we provide to patients is safe and effective, it remains vital to consider the patients foremost when managing infertility. As CAM is used so frequently (unsubstantiated claims of efficacy notwithstanding) there is a clear need for further research on this topic [3]. However, treatment offered for infertility should be patient, not doctor, centred. Patients should be treated holistically, respecting their own views and moral/ ethical framework. Our study suggests that CAM may be addressing a need that is not fully met by traditional medical practices.

## Competing interests

The author(s) declare that they have no competing interests.

## Authors' contributions

CC and JJJ contributed equally to this manuscript.

## Supplementary Material

Additional File 1 Questionnaire on Alternative or Complementary Medicine Click here for file
