# Supplementary material for: Complementary and alternative medicine utilisation in NHS and private clinic settings: a United Kingdom survey of 400 infertility patients
Source: J Exp Clin Assist Reprod. 2005 Apr 4;2:5. doi: 10.1186/1743-1050-2-5 (PMC1084360; doi:10.1186/1743-1050-2-5)
Supplement: Additional File 1 — Questionnaire on Alternative or Complementary Medicine [file 1743-1050-2-5-S1.doc]

Appendix 1. Questionnaire on Alternative or Complementary Medicine

I am trying to find out how popular alternative or complementary medicines are and would be very grateful if you would take a few moments to answer the following questions:

1. How old are you? Are you male or female?

2. Have you tried any of the following complementary therapies to help your fertility problem?

Please circle any you have tried:

Acupuncture

Traditional Chinese medicine

Medical herbalism

Reflexology

Hypnosis

Spiritual healing

Nutritional advice

Any other – please specify

None

3. Would you consider any complementary medicine in the future? YES / NO

If yes, do you know which one? ……………………………………………….

4. If you have tried any complementary medicine for your fertility problem, could you say whether it was helpful and how? YES / NO

………………………………………………..

5. Were you concerned about any possible side effects or risks attached to the treatment? YES / NO

………………………………………………..

6. Have you used any complementary medicine for any other medical problem in the past? YES / NO

………………………………………………..

7. Any further comments? ……………………………………………….
